# Two-Dimensional Decoupling and Decomposition Analysis of CO_2_ Emissions from Economic Growth: A Case Study of 57 Cities in the Yellow River Basin

**DOI:** 10.3390/ijerph191912503

**Published:** 2022-09-30

**Authors:** Yawen Kong, Chunyu Liu, Shuguang Liu, Shan Feng, Hongwei Zhou

**Affiliations:** 1 School of Economics, Ocean University of China, Qingdao 266100, China; 2 School of Oceanography, Shanghai Jiao Tong University, Shanghai 200030, China; 3 Institute of Marine Development, Ocean University of China, Qingdao 266100, China

**Keywords:** Yellow River Basin, energy-related carbon dioxide emissions, general two-dimensional decoupling model, LMDI method

## Abstract

Precise decoupling of CO_2_ emission and economic development holds promise for the sustainability of China in a post-industrialization era. This paper measures the energy-related CO_2_ emissions of 57 cities in the Yellow River Basin (YRB) during 2006–2019 and analyzes their decoupling states and dynamic evolution paths based on the derived general analytical framework of two-dimensional decoupling states to decompose their decoupling index using the LMDI method. The results show that (1) from 2006 to 2019, the economic growth and CO_2_ emissions of cities along the YRB are dominated by weak decoupling at an average contribution of 53.2%. Their dynamic evolution paths show fluctuations of “decoupling–recoupling” states, while the evolution trend is relatively ideal. (2) The factors of economic output, energy intensity and population scale inhibit the decoupling in most cities, which contribute 39.44%, 19.34%, and 2.75%, respectively, while the factors of industrial structure, carbon emission coefficient, and energy structure promote the decoupling in most cities in the YRB, with average contributions of −12.63%, −8.36%, and −0.67%, respectively. (3) The significant increase in the contribution of energy intensity is the main reason for the “Worse” path of cities, while the industrial structure and energy structure factors promote to the “Better” path of cities. This work satisfies the urgent need for the ecological protection of the YRB and opens new avenues for its high-quality development.

## 1. Introduction

Entering the epoch of industrialization and urbanization, the frequent energy extraction and utilization activities challenge the carrying capacity of the environment, causing excessive carbon dioxide emissions. The Sixth Assessment Report (AR6, 2021) of the Intergovernmental Panel on Climate Change (IPCC) clearly states that fossil fuel combustion is the underlying cause of the increasing greenhouse gas emissions, which severely impacts ecosystems, food security, and human health. If the Earth warms 2 more degrees, the complete melting of the Arctic ice floes and collapse of the Antarctic ice cap will potentially threaten marine corals, marine biodiversity, and human beings. Thus, the continuous global warming necessitates worldwide attention.

The United Nations (UN) has attached great importance to climate change, and a series of agreements have been promulgated since the 20th century. In 1992, the United Nations Framework Convention on Climate Change was adopted and entered into force with the signatures of 197 countries. The convention sets different emission reduction targets for countries at different levels of economic development and advocates that high-carbon-emitting developed countries take specific emission reduction measures and pay compensation for the extra emissions. However, quantifying the accurate carbon emission reduction target remains challenging and highly desired. Therefore, the Kyoto Protocol supplemented the quantified targets to control carbon emissions for developed countries, which alleviated the excessive carbon emissions. Subsequently, the Copenhagen Accord differentiated the carbon emission reduction tasks according to the total output of each country. In 2015, nearly 200 countries signed the Paris Agreement in order to keep the temperature rise below 2 degrees. The climate change conferences with worldwide agreements have played a great role in curbing global warming. Developing the low-carbon economy has become an international consensus.

As the world’s largest developing country, China faces huge carbon-reduction pressure from both domestic and international sources. According to the UN, China’s CO_2_ emissions have been growing at an average annual rate of 10% since 2000, up to 24.2% by 2010, making China the world’s highest total carbon emitter [1]. Besides, China’s consumption of energy increased nearly five times from 1978 to 2010, making it the world’s largest energy consumer. In response to climate change, China has constantly made voluntary commitments to reduce emissions. China managed to reduce CO_2_ emissions per unit of GDP by 48.4% in 2020 compared to 2005, and promised to reduce it down to 60%-65% by 2030 compared to 2005. At the UN General Assembly, China committed to reach carbon peak by 2030 and carbon neutrality by 2060, achieving a strong decoupling of economic growth, resource consumption, and carbon emissions [2,3]. To approach the targets above, the regions with key energy consumption need to be emphasized.

As a typical area for China, the Yellow River Basin (YRB) acts as an important ecological barrier and “energy basin” [4]. About 80% of China’s coal chemical enterprises are located in cities along the Yellow River, contributing 70% of the coal supply [5,6]. It is worth mentioning that the economic development of the YRB still relies highly on the coal chemical industry, leading to significant high carbon lock-in effects and contradictions between economic growth and environmental protection [7]. Therefore, the absolute decoupling of economic growth and carbon emissions will potentially dominate the sustainable development of the YRB. For this purpose, this paper systematically measures the energy-related CO_2_ emissions of cities in the YRB, dynamically monitors the decoupling level of carbon emissions from economic growth, and proposes a possible mechanism. The decoupling analysis and decomposition of CO_2_ emissions and economic growth provide theoretical support for formulating, implementing, and evaluating carbon-reduction strategies in the basin.

## 2. Literature Review

Currently, excessive CO_2_ emissions caused by human activities are accelerating global warming. A large body of literature has focused on the characteristics and drivers of CO_2_ emissions [8,9], the relationship of CO_2_ emissions and economic growth and its factors [10,11], and carbon-reduction paths [12]. Among them, the study of the relationship between CO_2_ emissions and economic growth has become a top topic. Scholars have used the environmental Kuznets curve (EKC) and the decoupling model to analyze their correlation and decoupling states, respectively [13,14]. In addition, the structural decomposition analysis (SDA) and exponential decomposition analysis (IDA) [15] combined with the extended STIRPAT model [16], as well as the Kaya equation [17], were used to decompose the factors in the relationship between carbon emissions and economic development.

The EKC was derived from the “inverted U-shaped” curve proposed by the American economist A. Kuznets in the 1950s, which depicts the evolution of per capita income with the process of economic development [18]. Subsequently, G. Grossman and A. Kureger (1991) extended it into the environmental area [19]. From then on, the EKC curve was widely used to verify the correlation between economic growth and environmental pollution and to predict their inflection point. For example, Selden and Song (1994) [20] demonstrated an inverted U-shaped EKC curve for global economic development and CO_2_ emissions, while Friedl and Getzner (2003) [21] and Martinez-Zarzoso et al. (2004) [13] proposed an N-shaped curve. Zhu (2014) [22] denoted that the EKC curves in the Bohai Sea Rim region had different forms according to the environmental quality indicators. Churchill et al. (2018) [23] revealed that nine countries had EKC curves from 1870–2014, exhibiting three forms: inverted U-shaped, N-shaped, and inverted N-shaped.

The Tapio decoupling model evaluates the decoupling state between economic growth and CO_2_ emissions [14]. Compared with the OECD decoupling model, this model refines the decoupling state criteria without standardizing scale. The current research of decoupling focuses on two aspects; one is to analyze the carbon decoupling states at the national, regional, and industry levels. For example, Wang et al. (2020) [24] compared the decoupling effects in developed and developing countries; unlike the developing countries, most developed countries showed a transition from a weak decoupling to a strong decoupling state. Zhang et al. (2022) [25] studied the carbon decoupling states of four regions in China and found that the decoupling states of the YRB were lower than those in the other three regions. Li et al. (2017) [26] noted that the construction industry showed various carbon decoupling relationships in different provinces. The other aspect is to explore the factors of decoupling between CO_2_ emissions and economic growth. For example, Wang et al. (2019) [27] showed that the energy-saving effect was the key factor to the carbon decoupling of the transportation industry in China. Ning et al. (2017) [28] found that energy-intensity factors contributed to the carbon decoupling in China’s Yangtze River Economic Zone. Zhao et al. (2018) [29] proposed that the main influencing factors for carbon decoupling in six major sectors were energy intensity and economic output.

In addition, some scholars improved the Tapio decoupling model by introducing the economic development level as an extra factor to construct a two-dimensional decoupling analysis framework of CO_2_ emissions and economic growth. For example, Song et al. (2019) [30] established the two-dimensional decoupling model by deriving the relationship equation between the decoupling model and the inverted EKC curve. Song et al. (2020) [31] constructed a Tapio-Z decoupling model to analyze the carbon decoupling states and their dynamic paths in China. Xin et al. (2021) [32] found that the two-dimensional decoupling state of Gansu province was in a weak decoupling–low level of economy (WD-LE) state from 2000–2017, and they proposed specific carbon reduction methods.

In summary, the existing literature has laid the foundation to further study the energy-related CO_2_ emissions and economic growth of cities in the YRB. However, the general decoupling framework between economic growth and CO_2_ emissions in cities of a certain basin remains challenging, but highly desired. Therefore, this paper constructs a general two-dimensional decoupling analysis framework of CO_2_ emissions and economic growth and studies the carbon decoupling of the YRB from the urban perspective. First, the CO_2_ emissions of 57 cities in the YRB are measured, and then the one- and two-dimensional decoupling states and their dynamic paths are well-analyzed. Finally, the carbon decoupling index is decomposed using the logarithmic mean Divisia index (LMDI) method to explore their inner mechanisms and to investigate the underlying reasons for the changes in the decoupling states of cities in the YRB. We provide theoretical support for the development of carbon-reduction policies in the YRB.

## 3. Material and Methods

### 3.1. CO_2_ Emission Accounting Model

This paper considers direct and indirect CO_2_ emissions of energy consumption. The direct carbon emissions ($Q_{1}$) mainly include natural gas and liquefied petroleum gas, while the indirect carbon emissions ($Q_{2}$) come from the urban power supply [33], heat supply, and transportation [34,35]. Considering the low share of urban-transport CO_2_ emissions [36], they are not included in this model. The method for measuring urban carbon emissions is shown in Equation (1).

(1) $$\begin{array}{ll} Q_{CO_{2}} & =Q_{1}+Q_{2}\\ & =\sum_{i}^{n}K_{i}\cdot E_{i}+\left(C_{e}\times EF_{y}+\frac{C_{h}}{A_{h}}\times E_{h}\times K_{c}\right) \end{array}$$

where $E_{i}$ and $K_{i}$ denote the urban energy consumption and CO_2_ emission coefficient of natural gas and liquefied petroleum gas, respectively. $C_{e}$ is the city’s electric energy consumption, and $EF_{y}$ is the CO_2_ emission factor of each regional grid. $C_{h}$ denotes the total amount of heat supply. $A_{h}$ denotes average low calorific capacity of raw coal. $E_{h}$ denotes the thermal efficiency of coal-fired industrial boilers. $K_{c}$ denotes the carbon emission factor of raw coal. The minimum standards for thermal efficiency of coal-fired industrial boilers are between 65–78%. Since the current central heating boilers in China are mainly small and medium-sized boilers, this paper takes the thermal efficiency of coal-fired industrial boilers $E_{h}$ as 70% [37].

### 3.2. Two-Dimensional Decoupling Model

#### 3.2.1. Environmental Kuznets Curve

The environmental Kuznets curve (EKC) describes the relationship between economic development and the level of environmental pollution of a country or a region. In recent years, scholars have confirmed that EKC curves exist in various forms, such as U-shaped [23], inverted U-shaped [38,39], N-shaped [26], and inverted N-shaped [40], so we define the functional equation of EKC curves as:

(2) $$\ln C_{it}=\alpha_{0}+\alpha_{1}\ln g_{it}+\alpha_{2}{\left(\ln g_{it}\right)}^{2}+\alpha_{3}{\left(\ln g_{it}\right)}^{3}+\lambda_{i}+\mu_{t}+\varepsilon_{it}$$

where $C_{it}$ and $g_{it}$ denote CO_2_ emissions and GDP per capita of city i in year t, respectively. $\alpha_{0}$ indicates the constant term. $\alpha_{1}$, $\alpha_{2}$, and $\alpha_{3}$ denote the coefficients of the primary, secondary, and tertiary terms, respectively. $\lambda_{i}$ and $\mu_{t}$ denote city and time fixed effect, respectively. $\varepsilon_{it}$ denotes the stochastic error term. The types of EKC curve corresponding to the values of the primary, secondary, and tertiary terms are shown in Table 1.

#### 3.2.2. Basic Decoupling Model

Referring to the decoupling elasticity index model proposed by Tapio, we set the carbon decoupling index model of cities in the YRB as follows:

(3) $$D_{C,G}=\frac{\%\Delta C/C_{i,t-1}}{\%\Delta G/G_{i,t-1}}=\frac{(C_{it}-C_{i,t-1})/C_{i,t-1}}{(G_{it}-G_{i,t-1})/G_{i,t-1}}$$

where $D_{C,G}$ denotes the Tapio decoupling elasticity index of city i in year t. $C_{it}$ and $C_{i,t-1}$ denote CO_2_ emissions of city i in year t and t−1, respectively. $G_{it}$ and $G_{i,t-1}$ denote GDP of city i in year t and t−1, respectively.

**Figure 1:**
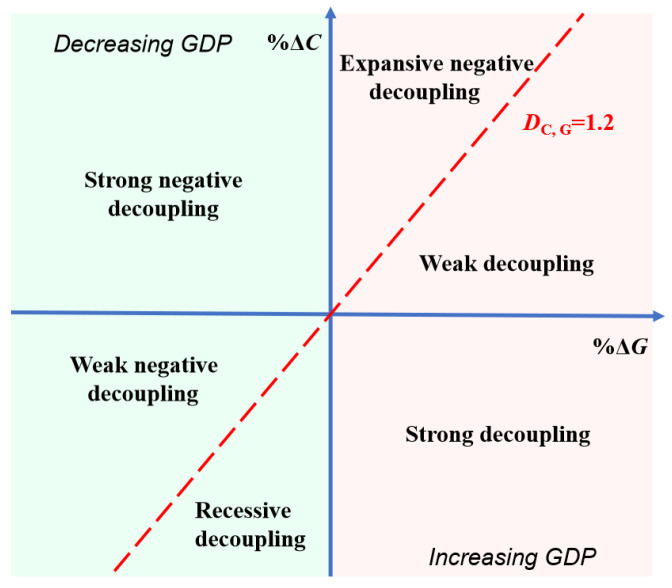
Classification of decoupling states.

Based on the classification criteria of the OECD and Tapio, we set six decoupling states as shown in Figure 1. (1) ΔG>0. If $D_{C,G}>1.2$, the city is in an expansive negative decoupling state (END), when the urban economy grows at the cost of increasing carbon emissions. If $0<D_{C,G}<1.2$, the city is in a weak decoupling state (WD), and the growth rate of urban economy is greater than that of carbon emissions. If $D_{C,G}<0$, the city is in a strong decoupling state (SD). The growth rate of urban economy increases while the amount of carbon emissions gradually declines, and the economic growth is freed from dependence on resource consumption. (2) ΔG<0. If $D_{C,G}<0$, the region is in the worst state of strong negative decoupling (SND). At this point, the urban GDP declines while the growth rate of carbon emissions increases. If $0<D_{C,G}<1.2$, the city is in a weak negative decoupling state (WND). The decreasing rate of urban GDP is greater than the rate of carbon reduction. If $D_{C,G}>1.2$, the region is in a recessive decoupling state (RD). The rate of economic decline is less than the rate of carbon reduction.

#### 3.2.3. Two-Dimensional Decoupling Model

Currently, the basic decoupling index model cannot distinguish the decoupling status of regions under different levels of economic development [30]. For example, regions with lower levels of economic development may reach a better decoupling state than regions with higher levels of economic development. To distinguish the decoupling state of regions with different levels of economic development, we characterize the economic development level by the GDP per capita indicator. We derive the relationship equation between the EKC curve equation and the carbon decoupling index model at different levels of economic development [31].

The EKC curve equation is approximated as a continuous function and expressed as:

(4) $$\ln C=\alpha_{0}+\alpha_{1}\ln g+\alpha_{2}{\left(\ln g\right)}^{2}+\alpha_{3}{\left(\ln g\right)}^{3}+\mu$$

We take the derivative of Equation (4), which can be expressed as Equations (5) and (6):

(5) $$\frac{dC}{dg}\cdot \frac{1}{C}=[\alpha_{1}+2\alpha_{2}\ln g+3\alpha_{3}{\left(\ln g\right)}^{2}]\cdot \frac{1}{g}$$

(6) $$\frac{dC/C}{dg/g}=\alpha_{1}+2\alpha_{2}\ln g+3\alpha_{3}{\left(\ln g\right)}^{2}$$

The Tapio decoupling index $D_{C,G}$ denotes the ratio of the rate of change of CO_2_ to that of GDP. We assume that the rate of change of the population is constant, that is, $p_{t}=p_{t-1}=p$, and take $D_{C,G}$ as a continuous function as follows:

(7) $$D_{C,G}=\frac{(C+\delta C-C)/C}{(G+\delta G-G)/G}=\frac{(C+\delta C-C)/C}{(gp+\delta gp-gp)/gp}=\frac{dC/C}{dg/g}$$

Based on Equation (6) and Equation (7), the relationship equation between decoupling index and per capita GDP can be expressed as:

(8) $$D_{C,G}=\alpha_{1}+2\alpha_{2}\ln g+3\alpha_{3}{\left(\ln g\right)}^{2}$$

Referring to the two-dimensional decoupling analysis framework of Song et al. (2020) [30], based on Figure 1, we construct a general two-dimensional decoupling analysis framework applicable to different EKC curve types (Table 2). The critical values for defining the decoupling state are 0 and 1.2. Substituting $D_{C,G}=0$ and $D_{C,G}=1.2$ into Equation (8), we obtain the corresponding GDP per capita values, which are considered as the critical values for determining the levels of regional economic growth, as shown in Table 2.

As shown in Table 2, we classify economic development into three levels: low economic development level (LE), middle economic development level (ME), and high economic development level (HE). For the U-shaped curve, the city is in LE when $0<g<g_{0}$, in ME when $g_{0}<g<g_{1.2}$, and in HE when $g>g_{1.2}$. For the inverted U-shaped curve, the city is in LE when $0<g<g_{1.2}$, in ME when $g_{1.2}<g<g_{0}$, and in HE when $g>g_{0}$. For the N-shaped curve, every critical value of the decoupling index corresponds to two values of per capita GDP. The value of per capita GDP is ranked from small to large as: $g_{1.2}^{L}<g_{0}^{L}<g_{0}^{R}<g_{1.2}^{R}$. We define the city as being in LE when $0<g<g_{0}^{L}$, in ME when $g_{0}^{L}<g<g_{1.2}^{R}$, and in HE when $g>g_{1.2}^{R}$. In the same way, for the inverted N-shaped curve, the value of per capita GDP is ranked from small to large as: $g_{0}^{R}<g_{1.2}^{R}<g_{1.2}^{L}<g_{0}^{L}$. Therefore, we define the city as being in LE when $0<g<g_{1.2}^{R}$, in ME when $g_{1.2}^{R}<g<g_{0}^{L}$, and in HE when $g>g_{0}^{L}$. Thus, the decoupling states can be divided into 18 categories. “HE-SD” is the most desirable two-dimensional decoupling state, indicating that the region is in a high level of economic development, with CO_2_ emissions declining when the economy grows. “SND-LE” is the least desirable two-dimensional decoupling state, which indicates that the urban economy is declining faster than carbon emissions.

### 3.3. Decomposition Model of Decoupling Index

Referring to existing studies, we extend the Kaya identical equation [41], combining the characteristics of CO_2_ emissions at the urban level in the YRB. 

At present, the development of China’s regional economy is still accompanied by a large consumption of energy, which leads to the excessive CO_2_ emissions. Thus, economic growth is a key driver to promote the growth of carbon emissions [42]. We select the GDP per capita indicator to indicate the economy output factor. Studies have shown that the demographic factor has a two-way effect on carbon-emission changes [43,44,45]. The population growth also brings about changes in the demand for energy consumption, so we choose the population scale indicator to denote the demographic factors. The “heavy” industrial structure, mainly secondary industry, is the main reason for the increase in regional CO_2_ emissions [46]. The YRB has a high proportion of extractive industries and primary manufacturing industries, and the overall industrial structure is “heavy” [47,48]. Therefore, we select the ratio of the second industrial increment to GDP to represent the industrial structure factor. Improving the clean use of energy is an important means to promote the decoupling of regional carbon emissions from economic growth [49,50], and the energy-intensity, energy-structure factors are closely related with the level of clean energy use; thus, we choose the ratio of the total energy consumption to the second industrial increment to indicate the power-intensity factor, and we choose the proportion of consumption of energy i in total energy consumption to represent the power-structure factor. We use the ratio of CO_2_ emissions emitted by energy i to consumption of energy i to denote the carbon emission coefficient factor [51] (see Table 3 below). Then the extended Kaya equation is represented as follows:

(9) $$\begin{array}{ll} C & =\sum_{i}^{4}P\times \frac{GDP}{P}\times \frac{SI}{GDP}\times \frac{EC}{SI}\times \frac{EC_{i}}{EC}\times \frac{C_{i}}{EC_{i}}\\ & =\sum_{i=1}^{4}P\times G\times I\times E\times E_{i}\times Q_{i} \end{array}$$

where C, P, GDP, SI, $EC_{i}$, EC, and $C_{i}$ represent CO_2_ emissions, the total population, gross domestic product, the second industrial increment, consumption of energy i, total energy consumption, and the CO_2_ emissions emitted by energy i of cities, respectively.

We decompose Equation (9) using the additive decomposition form of the LMDI decomposition proposed by B. W. Ang (2005) [43]; then the change in carbon emissions from the base period to year t can be decomposed as:

(10) $$\begin{array}{ll} \Delta C & =C_{t}-C_{0}\\ & =\sum_{i=1}^{4}P_{t}\times G_{t}\times I_{t}\times E_{t}\times E_{it}\times Q_{it}-\sum_{i=1}^{3}P_{0}\times G_{0}\times I_{0}\times E_{0}\times E_{i0}\times Q_{i0}\\ & =\Delta C_{P}+\Delta C_{G}+\Delta C_{I}+\Delta C_{E}+\Delta C_{Ei}+\Delta C_{Qi} \end{array}$$

(11) $$\begin{array}{l} \Delta C_{P}=\sum_{i=1}^{4}\frac{C_{t}-C_{0}}{\ln C_{t}-\ln C_{0}}\times \ln(\frac{C_{P}^{t}}{C_{P}^{0}})\\ \Delta C_{G}=\sum_{i=1}^{4}\frac{C_{t}-C_{0}}{\ln C_{t}-\ln C_{0}}\times \ln(\frac{C_{G}^{t}}{C_{G}^{0}})\;\\ \Delta C_{I}=\sum_{i=1}^{4}\frac{C_{t}-C_{0}}{\ln C_{t}-\ln C_{0}}\times \ln(\frac{C_{I}^{t}}{C_{I}^{0}})\\ \Delta C_{E}=\sum_{i=1}^{4}\frac{C_{t}-C_{0}}{\ln C_{t}-\ln C_{0}}\times \ln(\frac{C_{E}^{t}}{C_{E}^{0}})\\ \Delta C_{Ei}=\sum_{i=1}^{4}\frac{C_{t}-C_{0}}{\ln C_{t}-\ln C_{0}}\times \ln(\frac{C_{Ei}^{t}}{C_{Ei}^{0}})\\ \Delta C_{Qi}=\sum_{i=1}^{4}\frac{C_{t}-C_{0}}{\ln C_{t}-\ln C_{0}}\times \ln(\frac{C_{Qi}^{t}}{C_{Qi}^{0}}) \end{array}$$

where ΔC decomposes into 5 effects. $\Delta C_{P}$, $\Delta C_{G}$, $\Delta C_{I}$, $\Delta C_{E}$, $\Delta C_{Ei}$, and $\Delta C_{Qi}$ represents population scale effect, economic output effect, industrial structure effect, power intensity effect, power structure effect, and carbon emission coefficient effect, respectively.

According to Equations (3), (10), and (11), the decomposition model of carbon emission decoupling index can be expressed as follows:

(12) $$\begin{array}{l} D_{C,G}=\frac{\%\Delta C}{\%\Delta G}=\frac{(C_{t}-C_{0})/C_{0}}{(G_{t}-G_{0})/G_{0}}\\ =\frac{(\Delta C_{P}+\Delta C_{G}+\Delta C_{I}+\Delta C_{E}+\Delta C_{Ei}+\Delta C_{Qi})/C_{0}}{\Delta G_{t}/G_{0}}\\ =\frac{\Delta C_{P}/C_{0}}{\Delta G_{t}/G_{0}}+\frac{\Delta C_{G}/C_{0}}{\Delta G_{t}/G_{0}}+\frac{\Delta C_{I}/C_{0}}{\Delta G_{t}/G_{0}}+\frac{\Delta C_{E}/C_{0}}{\Delta G_{t}/G_{0}}+\frac{\Delta C_{Ei}/C_{0}}{\Delta G_{t}/G_{0}}+\frac{\Delta C_{Qi}/C_{0}}{\Delta G_{t}/G_{0}}\\ =D_{P}+D_{G}+D_{I}+D_{E}+D_{Ei}+D_{Qi} \end{array}$$

where the decoupling index $D_{C,G}$ can be decomposed into six sub-decoupling indices (D). $D_{P}$, $D_{G}$, $D_{I}$, $D_{E}$, $D_{Ei}$, and $D_{Qi}$ denote the sub-decoupling indices of population scale, economic output, industrial structure, power intensity, power structure, and carbon emission coefficient, respectively. When ΔG>0, the smaller the $D_{C,G}$, the better the decoupling state. At this time, if D<0, this means that the sub-decoupling indices positively promote the decoupling of carbon emissions from economic growth. On the contrary, when ΔG<0, the greater the $D_{C,G}$, the better the decoupling state. At this point, if D>0, the sub-decoupling indices positively promote the decoupling as well [52].

### 3.4. Research Area and Data Source

The Yellow River is known as the “Mother River” in China, with a total length of 5464 km and an area of about 750,000 km^2^, accounting for about 7.8% of China’s land area. It flows through nine provinces from west to east in Qinghai, Sichuan, Gansu, Ningxia, Shaanxi, Shanxi, Inner Mongolia, Henan, and Shandong provinces and 69 regions (states, leagues, and cities) [53]. The YRB has a complex and fragile ecological environment that covers ecological zones such as Sanjiangyuan and the Qilian Mountains, and it is an important ecological corridor and ecological barrier in China.

**Figure 2:**
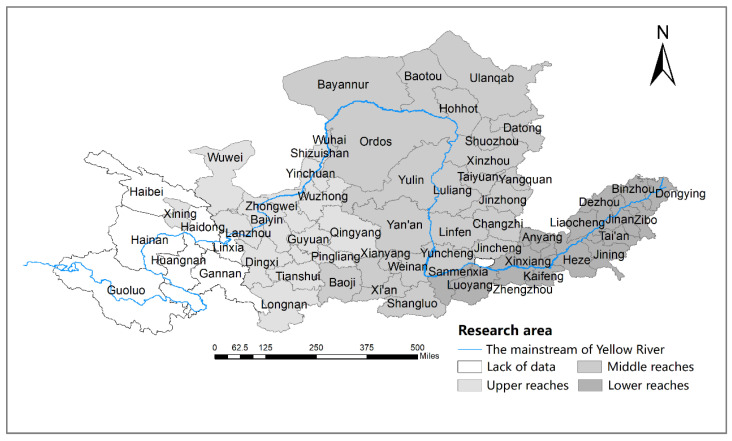
The research area.

This paper takes the prefecture-level cities and autonomous prefectures through which the main stream and the tributaries of the Yellow River flow as the study area. Due to the lack of data in Haidong city, Guoluo Tibetan Autonomous Prefecture, Huangnan Tibetan Autonomous Prefecture, Linxia Hui Autonomous Prefecture, Haibei Tibetan Autonomous Prefecture, Gannan Tibetan Autonomous Prefecture, Hainan Tibetan Autonomous Prefecture, and Jiyuan City, and the incorporation of Laiwu into Jinan City, the above-mentioned areas were excluded. The study area was finally determined as 57 prefecture-level cities where the Yellow River flows through eight provinces (see Figure 2 below). 

The data used in this paper are mainly divided into two parts: (1) CO_2_ emission accounting data. The data on energy consumption such as natural gas and liquefied petroleum gas, total electricity consumption, and heat supply of 57 cities in the YRB from 2006–2019 are from the *China City Statistical Yearbook, 2007–2020*. The CO_2_ emission coefficients in 2006–2019 are from *the General Rules for Calculating Comprehensive Energy Consumption* and *Guidelines for Preparing Provincial Greenhouse Gas Inventories* (Table 4). The CO_2_ emission factors of each regional grid are from China’s grid (Table 5 and Table 6). Linear interpolation was used to deal with missing data in individual years. (2) Socio-economic data: the GDP and GDP per capita of 57 cities in the YRB from 2006–2019 are obtained from every provincial statistical yearbook from 2007–2020 and converted to constant 2006 prices.

## 4. Results

### 4.1. Decoupling Analysis

#### 4.1.1. Basic Decoupling Analysis

**Figure 3:**
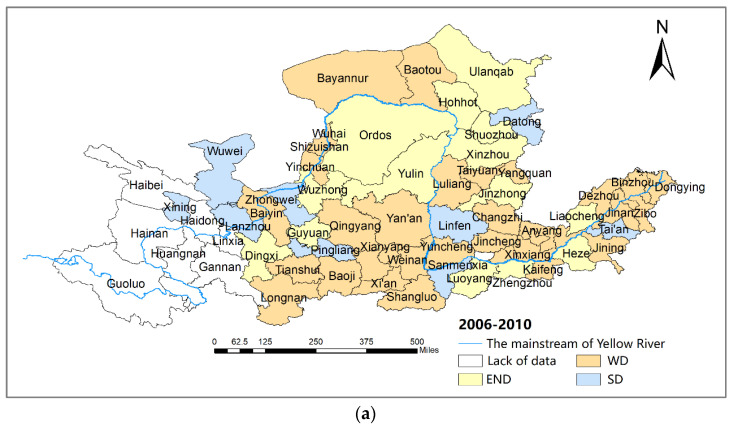

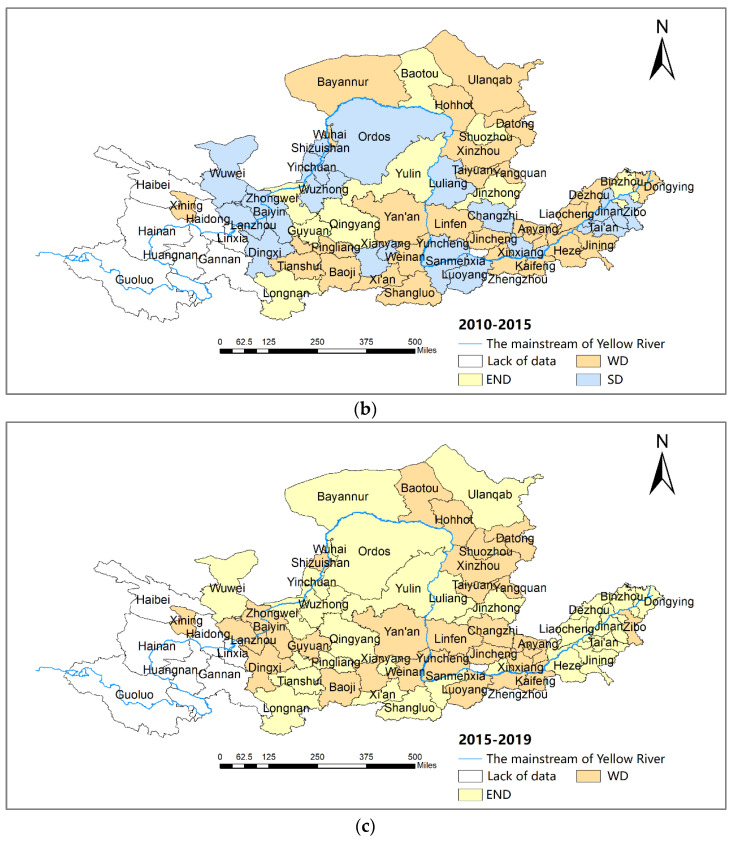
Decoupling states of cities in the YRB, 2006–2019. (**a**) Decoupling states of cities, 2006–2010. (**b**) Decoupling states of cities, 2010–2015. (**c**) Decoupling states of cities, 2015–2019.

According to the Tapio decoupling elasticity index model presented in Equation (7), Figure 3 shows the decoupling states from CO_2_ emissions and economic development of 57 cities in 2006–2010, 2010–2015, and 2015–2019. The carbon decoupling states of 57 cities in the YRB change significantly and appear as three types, expansion-negative decoupling (END), weak decoupling (WD), and strong decoupling (SD), during the study period. The WD state dominates the states at an average contribution of 53.2%.

Specifically, in 2006–2010, cities in the YRB showed a spatio-temporal distribution of “WD and END states in the upper reaches, and WD state in the middle and lower reaches”. About 59.6% of the cities were in a WD state when the growth rate of economic development is higher than that of carbon emissions; 24.6% of the cities were in an END state when economic growth depends on the increase in carbon emission; and 15.8% of the cities were in an SD state when economic growth is free from resource dependence. That is, only 15.8% of cities had the desired decoupling states during the period. The proper reason is that the heavy and chemical industries have been the main growth points of the economy in the YRB; meanwhile, the “heavy” industrial structure and lack of effective carbon-reduction measures of cities in the YRB lead to a rapid increasing of carbon emissions. 

In 2010–2015, the decoupling states of cities in the YRB transformed into a pattern of “SD state in the upper reaches and WD state in the middle and lower reaches”. The proportion of the dominant decoupling type, the WD state, decreased to 50.9% in cities in the YRB. Cities of the END state decreased by 8.8%, while cities of the SD state increased by one time, accounting for 33.3%. The trend of the decoupling state improved compared to the previous period. A probable explanation is that under the background of the low-carbon economy transition proposed by China, cities in the YRB actively carried out energy conservation and emission reduction by energy restructuring, industrial structure optimization, and control of total energy consumption. In addition, cities of the YRB developed clean energy such as hydropower and wind power according to the local natural endowments; thus, the carbon decoupling states of the cities improved in the period. 

In 2015–2019, the spatio-temporal distribution pattern of cities in the YRB transformed into “WD and END states in the upper and lower reaches, and WD state in the middle reaches”. The dominant decoupling type changes to the END and WD states. The decoupling states of cities in this stage was relatively less desirable than in the previous stage. The possible reason is that with the shift to high-quality development of the economy, cities in the YRB carried out a series of rectifications of high-energy-consuming enterprises in the key areas. The rectifications caused a significant reduction in traditional kinetic energy, but new economic growth points were not yet completely cultivated at the same time.

#### 4.1.2. Two-Dimensional Decoupling Analysis

**Figure 4:**
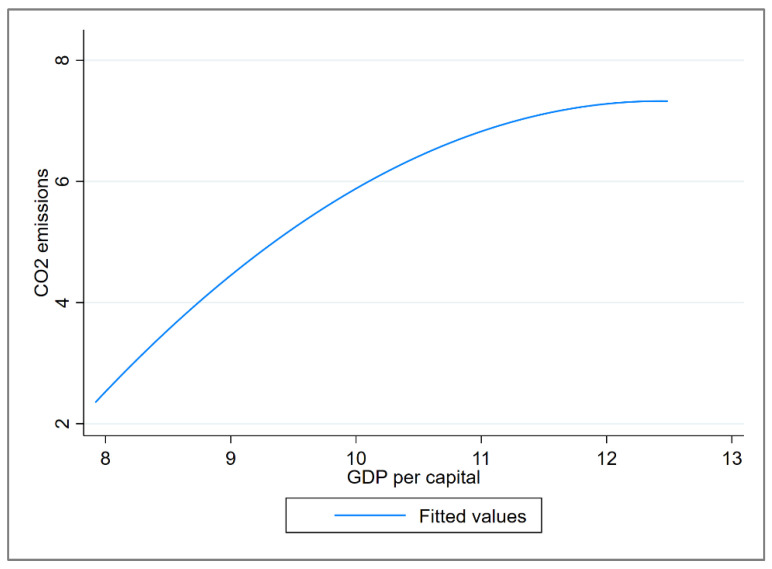
The fitting curve of CO_2_ emissions and per capital GDP, 2006–2019.

Based on the EKC curve of Equation (8), Figure 4 and Table 7 identify the regression results. All variables are significant at a 5% significance level. Figure 4 shows an inverted curve of carbon emissions and real GDP per capita of cities along the YRB. According to the $g_{0}=e^{-\frac{\alpha_{1}}{2\alpha_{2}}}$ and $g_{1.2}=e^{\frac{1.2-\alpha_{1}}{2\alpha_{2}}}$ equations in Table 2, the threshold values to measure the level of economic development are obtained as RMB 10,269 and RMB 40,854.

**Figure 5:**
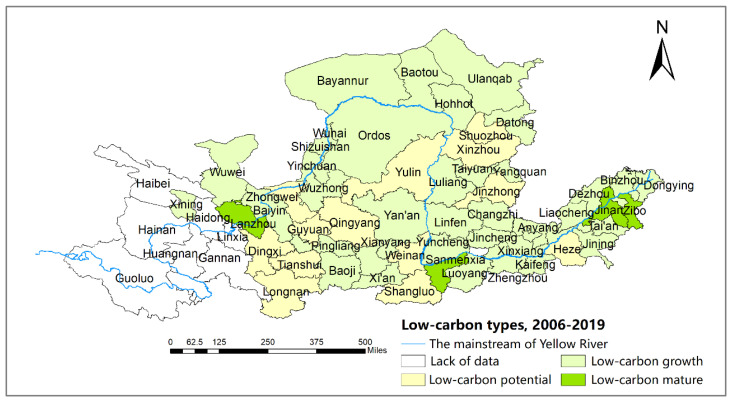
Low-carbon types of cities in the YRB by total score, 2006–2019.

**Figure 6:**
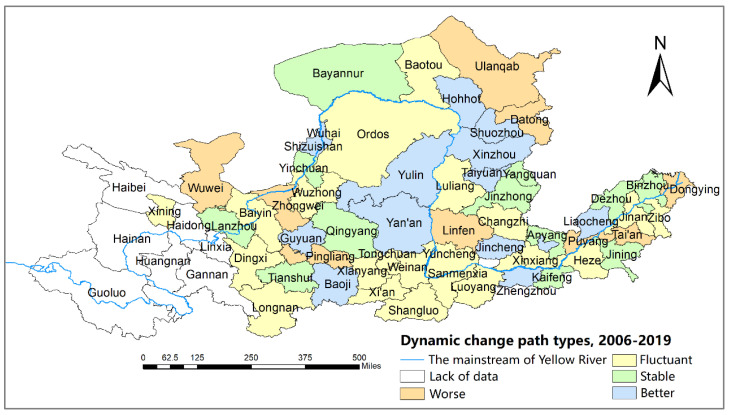
Dynamic change path types for two-dimensional decoupling states, 2006–2019.

According to the classification of the two-dimensional decoupling states presented in 3.2.3, Table 8 indicates the two-dimensional decoupling states, dynamic path, and scores of 57 cities. The cities in the YRB are classified into Type I: low-carbon mature (total score: 9–12), Type II: low-carbon growth (total score: 5–8), and Type III: low-carbon potential (total score: 1–4). Besides, cities are classified into four categories (better, stable, fluctuant, and worse) based on the dynamic paths of the two-dimensional decoupling states. The “better” type expresses an increasing scores of two-dimensional decoupling states in three time periods, and the “worse” type is vice versa. The “stable” type denotes the same score of two-dimensional decoupling states over three time periods. The “fluctuant” type represents the fluctuating score of two-dimensional decoupling states in three time periods. The results are shown in Table 8, Figure 5 and Figure 6.

As shown in Table 8, “WD-ME” are the dominant two-dimensional decoupling states for cities along the YRB in 2006–2019, while cities in “END-HE” and “END-ME” increase significantly and become the vital decoupling states of cities in 2015–2019. Specifically, in 2006–2010, 12.3% of cities are in the “SD-ME” state where their economies are at a medium level of development, achieving economic growth while decreasing total carbon emissions. In total, 42.1% of cities are in the “WD-ME” state. Only Xinzhou, Dingxi, Heze, and Guyuan cities are in the less desirable “END-LE” state, whose economic development level is low and economic growth is more dependent on energy consumption. In 2010–2015, Zibo, Sanmenxia, Jinan, Ordos, Luoyang, and Yinchuan cities were in the most desirable “SD-HE” states. The “WD-ME” states remain the dominant decoupling type for cities but with a slight decrease compared to the previous period. In 2015–2019, the cities of the ME increased from 15 to 28, while the cities in the “END-HE” state increased from 2 to 15, indicating that the jump in the economic development levels of some cities came at the cost of energy consumption.

The total score in Table 8 reflects that most cities belong to the low-carbon growth type (Type II), 13 cities are in the low-carbon potential type (Type III), and only 4 cities are in the low-carbon mature type (Type I). Figure 5 shows that cities of the low-carbon potential type (Type III) are mainly located in the middle reaches of the Yellow River. The proper reason is that these cities, which initially developed with abundant energy, had a low level of economic development. Zibo, Lanzhou, Sanmenxia, and Jinan cities, which are the low-carbon mature type, retain high total scores because of their medium and high economic development levels, as well as the SD and WD decoupling states in three periods. The increasing score of most cities in the low-carbon growth type (Type II) benefits from the jump in level of economic development. For example, Taiyuan and Jincheng cities achieved a jump from a medium to a high level of economic development while keeping the WD state unchanged.

Figure 5 identifies that the decoupling path of cities shows a repeated fluctuation of decoupling–recoupling. Most cities are the “fluctuant” type, followed by the “better” and “stable” types, and only nine cities are consistently the “worse” type. The reason is that cities with relatively ideal decoupling paths have carried out energy conservation and industrial transformation in the process of economic growth. However, the decoupling paths fluctuate because of the different timing and implementation effects of energy-conservation and emission-reduction policies in different cities. Specifically, although the total scores of “III-better” type cities such as Shuozhou, Yulin, and Guyuan are low, these cities have taken measures to increase the proportion of clean-energy use and promote industrial transformation to gradually reduce their requirements on traditional energy sources while enhancing their economic development. With little improvement in the level of economic development, the decoupling states of Wuhai, Dongying, Tai’an, Wuwei, Datong, Linfen, Puyang, and Zhongwei cities continue to deteriorate from the SD and WD states to the END state. Among them, Zhongwei city, a typical representative of depending on energy consumption to promote economic growth, has the worst decoupling situation as a “III-worse” type. Tianshui, Qingyang, and Jinzhong cities are in the “III-stable” type, when the level of economic development improves but the decoupling state becomes worse. These cities need to further promote energy-structure and industrial-structure adjustment and reduce the proportion of heavy chemical industries and high-energy-consuming industries to reduce CO_2_ emissions.

To sum up, the decoupling of economic growth and carbon emissions in cities along the YRB in 2006–2019 has not yet reached an ideal state. During the study period, the economic development of cities in the YRB had not been decoupled from energy consumption. Therefore, it is necessary to analyze the drivers of decoupling to better realize the low-carbon and sustainable development of cities in the YRB. 

### 4.2. Driving Factors Analysis

**Figure 7:**
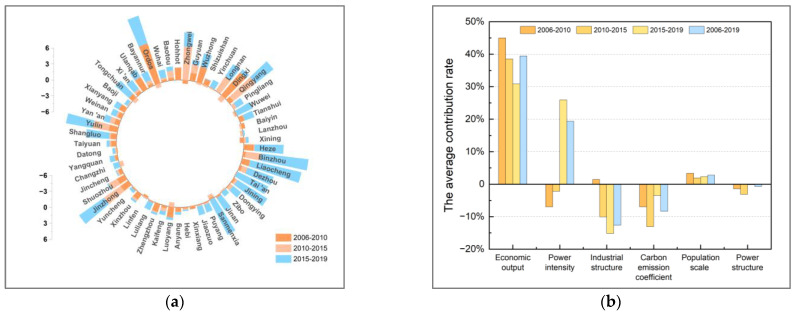
Cumulative contribution value and cumulative contribution rate in 2006–2010, 2010–2015, and 2015–2019. (**a**) Cumulative contribution value (decoupling index). (**b**) Cumulative contribution rate of six factors.

**Figure 8:**
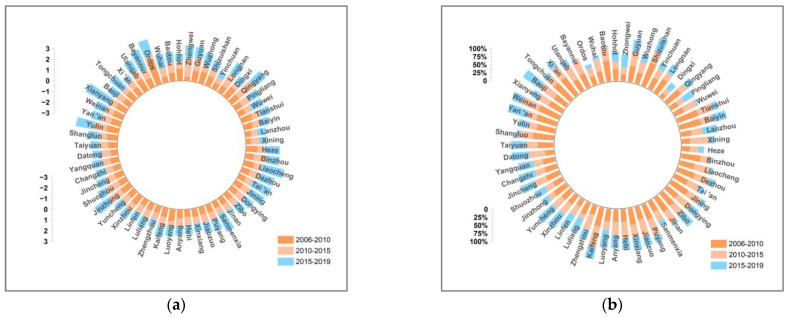
Contribution and contribution rate of economic-output factor. (**a**) Contribution of economic-output factor. (**b**) Contribution rate of economic-output factor.

**Figure 9:**
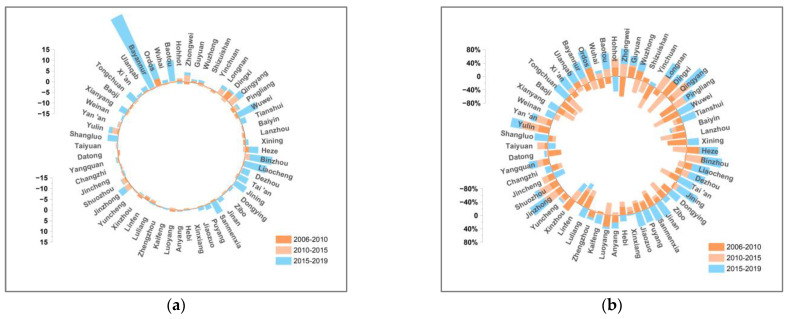
Contribution and contribution rate of power-intensity factor. (**a**) Contribution of power-intensity factor. (**b**) Contribution rate of power-intensity factor.

**Figure 10:**
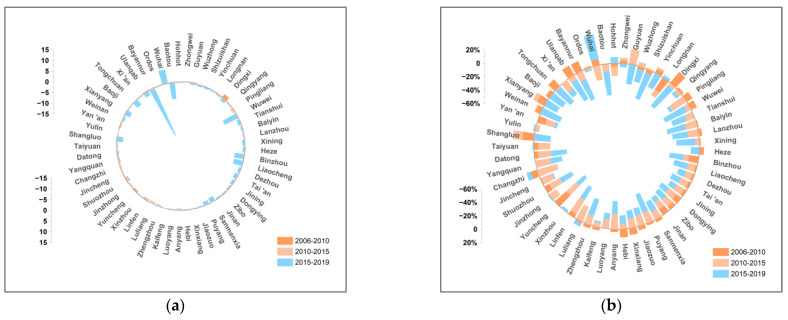
Contribution and contribution rate of industrial-structure factor. (**a**) Contribution of industrial-structure factor. (**b**) Contribution rate of industrial-structure factor.

**Figure 11:**
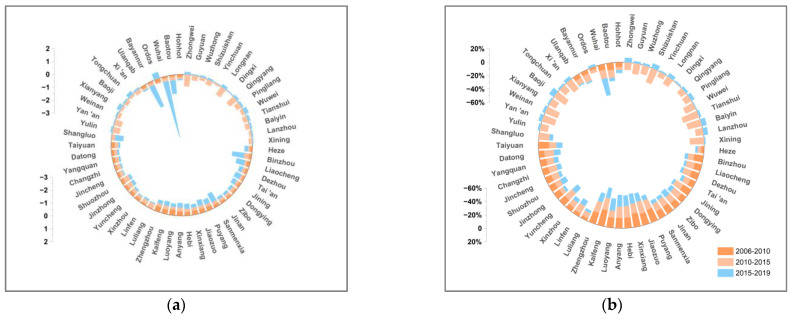
Contribution and contribution rate of carbon-emission-coefficient factor. (**a**) Contribution of carbon-emission-coefficient factor. (**b**) Contribution rate of carbon-emission-coefficient factor.

**Figure 12:**
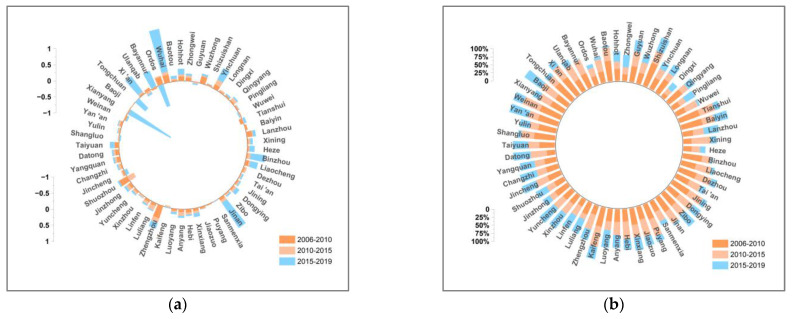
Contribution and contribution rate of population-scale factor. (**a**) Contribution of population-scale factor. (**b**) Contribution rate of population-scale factor.

**Figure 13:**
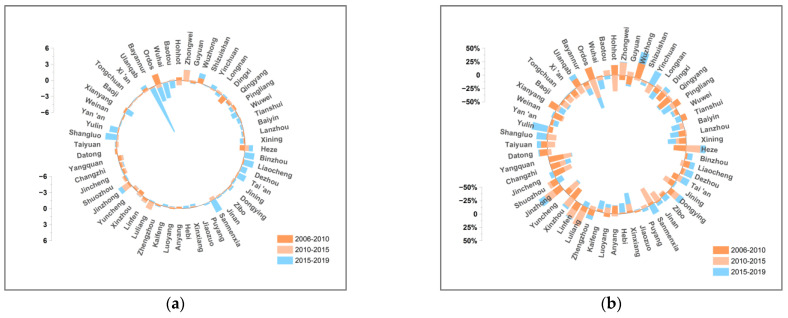
Contribution and contribution rate of power-structure factor. (**a**) Contribution of power-structure factor. (**b**) Contribution rate of power-structure factor.

This paper decomposes the decoupling indexes of 57 cities in the YRB using the LMDI additive model in 2006–2010, 2010–2015, and 2015–2019. Figure 7, Figure 8, Figure 9, Figure 10, Figure 11, Figure 12 and Figure 13 show the contribution values and relative contribution rates of energy-structure, energy-intensity, industrial-structure, economic-output, and population-scale factors to the decoupling index. The absolute magnitude of the contribution value of each factor represents its influence on decoupling, with positive values representing the factor’s inhibitory effect on decoupling and negative values representing the factor’s facilitative effect on decoupling. The relative contribution rate of a factor is the ratio of its absolute value of sub-decoupling index value (contribution value) to the sum of the absolute values of each factor’s sub-decoupling index.

As shown in Figure 7b, the six factors have significant differences in the contribution of the decoupling of 57 cities in the YRB from 2006 to 2019. Ranked from high to low by the average cumulative contribution are the economic-output, energy-intensity, industrial-structure, carbon-emission-coefficient, population-scale, and energy-structure factors, respectively, whose contribution to decoupling are 39.44%, 19.34%, −12.63%, −8.36%, 2.75%, and −0.67%, respectively. Specifically, the economic-output, energy-intensity, and population-scale sub-decoupling indices are positive for most cities, indicating that they have a suppressive effect on the decoupling between CO_2_ emissions and economic growth in cities in the YRB. The industrial-structure, carbon-emission-coefficient, and energy-structure sub-decoupling indices are negative for most cities, indicating that they have a facilitating effect on the decoupling in the YRB cities.

From the perspective of the dynamic path of decoupling states in cities, the significant increase in the contribution rate of the energy-intensity factor is the main reason for cities to show the “worse” path of decoupling. For example, the contribution rates of the energy-intensity factor in Wuwei and Zhongwei cities rose from −50.85% and −59.66% in 2006–2010 to 50.73% and 45.71% in 2015–2019, respectively, leading to worse two-dimensional decoupling states. The decrease in the contribution of the energy-intensity factor is the most important reason for some cities to show a “better” path, such as Hohhot and Xinzhou cities in the “better” type; their contribution rate of the energy-intensity factor to the decoupling decreased from 27.02% and 36.66% to −44.99% and −27.22%, respectively. The industrial-structure factor and energy-structure factor also contributed to the “better” path of cities.

#### 4.2.1. Economic-Output Factor

The economic-output factor is the dominant factor inhibiting the decoupling of economic growth and CO_2_ emissions in cities in the YRB. Figure 8 shows that the economic-output sub-decoupling index of cities in the YRB is positive in three periods, and the economic-output decoupling index of most cities show a slow fluctuating decreasing trend, which indicates that the economic development of cities along the YRB has a hindering effect on the decoupling, but this gradually decreased. The reasonable explanation is that China has accelerated the transformation of the economic development mode since 2010 and entered the stage of high-quality development in 2017, which caused the economic growth rate to gradually decrease and stabilize. The EKC curve above in Figure 4 indicates that the “inflection point” of the relationship between economic growth and CO_2_ emissions has not yet appeared in most cities in the YRB. It is expected that the inhibitory effect of economic output on decoupling will further weaken and cross the inflection point.

#### 4.2.2. Energy-Intensity Factor

Energy intensity is a prominent factor inhibiting the decoupling of economic growth and CO_2_ emissions in most cities of the YRB. As shown in Figure 9, the contribution of the energy-intensity factor to the decoupling in cities of the YRB shows phase changes. In 2006–2010, the energy-intensity factor had a facilitating effect on decoupling for more than half of the cities with an average contribution rate of −6.98%. The change indicates that the energy efficiency of cities in the YRB improved during the Eleventh Five-Year Plan. In 2010–2015, the energy-intensity factor in most cities still had a positive effect on the decoupling, but its contribution diminishes, shifting to an average contribution rate of −2.19%. In 2015–2019, the energy-intensity sub-decoupling index shifts from negative to positive for most cities, and energy intensity becomes an important factor inhibiting the decoupling in most cities. Figure 7b identifies that energy intensity is the second key factor limiting the decoupling in cities in the YRB. Therefore, local governments of cities still need to focus on reducing carbon-emission intensity and continuously improve energy efficiency to promote the transformation of the energy-intensity factor into a major contributor to the sustainable decoupling of the YRB in the future.

#### 4.2.3. Industrial-Structure Factor

Industrial structure is one of the factors contributing to the decoupling of economic growth and carbon dioxide emissions in most cities in the YRB. As shown in Figure 10, the industrial-structure sub-decoupling index has experienced changes from positive to negative. In 2006 to 2010, the industrial-structure factor had a slight hindering effect on the decoupling in more than half of the cities, and the average contribution rate of the urban industrial structure to decoupling is only 1.46%. In 2010–2015, the industrial-structure sub-decoupling index of most cities changed from positive to negative. The industrial-structure factor shifted from inhibiting to promoting the decoupling with an average contribution rate of −10.08%. In 2015–2019, the industrial-structure factor made further improvements toward promoting the decoupling of almost all cities, with an average contribution rate of −15.18%. The reason for this change is probably that the industrial structure in cities of the YRB dominated by heavy chemical industries has been gradually adjusted and optimized. Since China entered the throes of economic structural adjustment in 2010, the government has formulated and implemented a series of special policies on energy conservation and emission reduction, such as the elimination of backward excess capacity, leading to the reduction in “weight” in the industrial structure. Therefore, the influence of the industrial structure on the decoupling of cities in the YRB increased more and more significantly.

#### 4.2.4. Carbon-Emission-Coefficient Factor

The carbon emission coefficient is one of the factors that promote the decoupling of economic growth and CO_2_ emissions in most cities in the YRB. The available calculation results show that the change of the carbon emission coefficient reflects the clean utilization level of energy [51]. Figure 11 shows that the contribution of the carbon-emission-coefficient factor to the decoupling in cities in the YRB varies between positive and negative. In 2006–2015, the carbon-emission-coefficient factor promoted the decoupling in cities with an average contribution rate of −10.03%. In 2015–2019, the overall changes of the carbon-emission-coefficient factor provided a decreasing promoting effect to the decoupling in cities with an average contribution rate that dropped to −3.49%. The immaturity of the clean utilization technologies in some cities may have caused this change. Local governments need to strengthen the investment in and R&D of clean energy utilization technologies in the future, and make full use of the abundant local wind, water, and solar energies for power generation to reduce the proportion of traditional energy input and improve the clean utilization of energy.

#### 4.2.5. Population-Scale Factor

The population scale is one of the factors that inhibit the decoupling of economic growth and CO_2_ emissions of most cities in the YRB. As shown in Figure 12, the population-scale sub-decoupling index is positive in most cities of the YRB with the average contribution rate to decoupling staying below 4%. This indicates that the population-scale factor slightly inhibits the decoupling, and its contribution rate is relatively stable. The available data show that the increase in resident population in most cities in the YRB from 2006 to 2019 is small and stable. However, with the implementation of China’s “two-child” and “three-child” family planning policies, population growth is expected to increase in the future. Therefore, the government needs to pay attention to controlling the increase in the population scale reasonably, so that the impact of the population scale on decoupling can be controlled in an appropriate range.

#### 4.2.6. Energy-Structure Factor

The energy structure is one of the factors contributing to the decoupling of economic growth and CO_2_ emissions in most cities in the YRB. Figure 13 shows that the energy-structure sub-decoupling index is negative in most cities of the YRB in three periods, while its contribution rate is relatively small, indicating that the energy structure plays a limited facilitating role in the decoupling of cities in the YRB. During the study period, the contribution rate of the energy structure showed a trend of increasing and then decreasing. The average contribution rate of the energy structure to decoupling was only −1.43% in 2006–2010, and its effect on decoupling decreased in 2010–2015 with an average contribution rate of −3.15%. The contribution rate of the energy-structure factor increased in 2015–2019 to −0.19%. This indicates that under the guidance of the new green development concept, cities in the YRB are actively exploring the path of clean energy to replace traditional energy. However, an energy structure dominated by fossil energy such as coal and oil is difficult to change in the short term; thus, the contribution of the energy structure to decoupling is very limited. In the future, cities in the YRB need to increase the proportion of clean-energy use and vigorously develop a new energy economy to reduce the reliance on traditional energy sources, so as to improve the energy structure’s effect on the promotion of decoupling.

## 5. Discussions

### 5.1. Revisiting Decoupling and Decomposition Analysis of Cities in the YRB

Absolute decoupling of CO_2_ emissions and economic development contribute to sustainability of China. A number of studies discuss the decoupling and decomposition of CO_2_ emissions and economic growth from national, provincial and industry and city level [25,26,27,28]. Some research considers the economic development level and introduces it into the analysis framework of decoupling that applicable for the inverted EKC curve [54]. The general decoupling framework of CO_2_ emissions and economic growth in cities of a certain basin remains challenging. Thus, this paper relaxes the restriction for the EKC curve and derives a general analysis framework for two-dimensional decoupling states. Besides, this paper takes 57 cities in the YRB as study area to measure their energy-related CO_2_ emissions during 2006–2019, analyze the one- and two-dimensional decoupling states of the cities and decompose the factors affecting the decoupling index using the LMDI method. This work improves the applicability of analysis framework for two-dimensional decoupling states and enriches the existing research scale. The results can provide theorical support for decision makers.

### 5.2. Limitations and Potential Solutions

In this paper, we illustrate the changes of one- and two-dimensional decoupling states in the YRB, decompose decoupling index using the LMDI method, and explore the inner mechanism of the change of the decoupling index. However, due to the lack of official data in some cities, energy types covered in this paper are not comprehensive, resulting in small CO_2_ emissions as a whole. We will improve the CO_2_ emission accounting model, use night light data and pay attention to the latest official datasets in future studies. In addition, this paper focuses on the cities along the YRB. Comparative studies on the decoupling states of international basins such as the Rhine River Basin and domestic basins such as the Yangtze River Basin in China can be added in the future.

## 6. Conclusions

This paper creatively constructed the general two-dimensional decoupling analysis framework to explore the dynamic evolution paths of decoupling states and detects the drivers of decoupling. This work will provide guidance for sustainable development of cities in the YRB. It is found that the decoupling states of cities are unstable with repeated fluctuations of “decoupling–recoupling” during 2006-2019. The transition from “WD-ME” to “END-HE” and “END-ME” indicates that the leap in economic development of some cities in the YRB comes at the cost of energy consumption. Those cities need to change their crude development mode that is based on heavy chemical industries. Besides, most cities belong to the low-carbon growth type (Type II), and their dynamic paths of the decoupling are fluctuating while relatively ideal. Finally, the economic output, energy intensity and population scale factors inhibit the decoupling in most cities in the YRB, while the industrial structure, carbon emission coefficient and energy structure factors promote the decoupling. The significant increase in the contribution of energy intensity is the main reason for the “worse” path of cities. The industrial structure and energy structure factors play a positive role in the “better” path of cities.

## Figures and Tables

**Table 1:** The relationship between coefficient values and EKC curves.

Primary Term	Secondary Term	Tertiary Term	Type
$\alpha_{1}<0$	$\alpha_{2}>0$	$\alpha_{3}=0$	U-shaped
$\alpha_{1}>0$	$\alpha_{2}<0$	$\alpha_{3}=0$	Inverted U-shaped
$\alpha_{1}>0$	$\alpha_{2}<0$	$\alpha_{3}>0$	N-shaped
$\alpha_{1}<0$	$\alpha_{2}>0$	$\alpha_{3}<0$	Inverted N-shaped

**Table 2:** A general two-dimensional decoupling analysis framework.

Curve Types	$D_{C,G}$	Per Capita GDP	Two-Dimensional Decoupling Analysis Framework
U-shaped	0	$g_{0}=e^{-\frac{\alpha_{1}}{2\alpha_{2}}}$	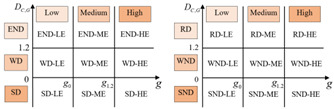 (a) ΔG>0 (b) ΔG<0
	1.2	$g_{1.2}=e^{\frac{1.2-\alpha_{1}}{2\alpha_{2}}}$	
Inverted U-shaped	0	$g_{0}=e^{-\frac{\alpha_{1}}{2\alpha_{2}}}$	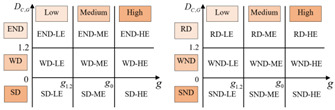 (c) ΔG>0 (d) ΔG<0
	1.2	$g_{1.2}=e^{\frac{1.2-\alpha_{1}}{2\alpha_{2}}}$	
N-shaped	0	$g_{0}^{L}=e^{-\frac{\alpha_{2}}{3\alpha_{3}}-\sqrt{\frac{\alpha_{2}{}^{2}-3\alpha_{1}\alpha_{3}}{9\alpha_{3}{}^{2}}}}$ $g_{0}^{R}=e^{-\frac{\alpha_{2}}{3\alpha_{3}}+\sqrt{\frac{\alpha_{2}{}^{2}-3\alpha_{1}\alpha_{3}}{9\alpha_{3}{}^{2}}}}$	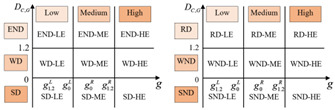 (e) ΔG>0 (f) ΔG<0
	1.2	$g_{1.2}^{L}=e^{-\frac{\alpha_{2}}{3\alpha_{3}}-\sqrt{\frac{\alpha_{2}{}^{2}-3\alpha_{1}\alpha_{3}+3.6\alpha_{3}}{9\alpha_{3}{}^{2}}}}$ $g_{1.2}^{R}=e^{-\frac{\alpha_{2}}{3\alpha_{3}}+\sqrt{\frac{\alpha_{2}{}^{2}-3\alpha_{1}\alpha_{3}+3.6\alpha_{3}}{9\alpha_{3}{}^{2}}}}$	
Inverted N-shaped	0	$g_{0}^{L}=e^{-\frac{\alpha_{2}}{3\alpha_{3}}-\sqrt{\frac{\alpha_{2}{}^{2}-3\alpha_{1}\alpha_{3}}{9\alpha_{3}{}^{2}}}}$ $g_{0}^{R}=e^{-\frac{\alpha_{2}}{3\alpha_{3}}+\sqrt{\frac{\alpha_{2}{}^{2}-3\alpha_{1}\alpha_{3}}{9\alpha_{3}{}^{2}}}}$	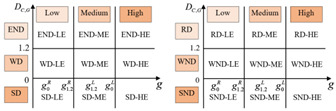 (g) ΔG>0 (h) ΔG<0
	1.2	$g_{1.2}^{L}=e^{-\frac{\alpha_{2}}{3\alpha_{3}}-\sqrt{\frac{\alpha_{2}{}^{2}-3\alpha_{1}\alpha_{3}+3.6\alpha_{3}}{9\alpha_{3}{}^{2}}}}$ $g_{1.2}^{R}=e^{-\frac{\alpha_{2}}{3\alpha_{3}}+\sqrt{\frac{\alpha_{2}{}^{2}-3\alpha_{1}\alpha_{3}+3.6\alpha_{3}}{9\alpha_{3}{}^{2}}}}$	

Notes. We assign appropriate values to the nine decoupling states. (a) When ΔG>0, SD-HE is the most ideal state for decoupling, let SD-HE be 4, and let SD-ME and SD-LE be 3 and 2, respectively. Similarly, let WD-HE, WD-ME, and WD-LE be 3, 2, and 1, respectively. Let END-HE, END-ME, and END-LE be 2, 1, and 0, respectively. (b) When ΔG<0, RD-HE is a relatively ideal decoupling state, and let it be 3, and let RD-ME and RD-LE be 2 and 1, respectively. Let WND-HE, WND-ME, and WND-LE be 2, 1, and 0. Let SND-HE, SND-ME, and SND-LE be 1, 0, and −1, respectively.

**Table 3:** Variables influencing decoupling.

Variables	Indicator Explanation	Symbol
Population scale	Resident population at year-end	P
Economic output	GDP per capita	G
Industrial structure	Ratio of the second industrial increment to GDP	I
Power intensity	Ratio of the total energy consumption to the second industrial increment	E
Power structure	The proportion of consumption of energy i in total energy consumption	$E_{i}$
Carbon emission coefficient	Ratio of CO_2_ emission emitted by energy i to consumption of energy i	$Q_{i}$

**Table 4:** Conversion factors of standard coal and CO_2_ emission factors.

Energy Types	Average Low Calorific Capacity (kJ/kg)	Conversion Factor of Standard Coal (kgce/kg, m^3^)	CO_2_ Content per Unit Calorific Value (t CO_2_/TJ)	Oxidation Rate of CO_2_	CO_2_ Emission Factor (kgCO_2_/kg)
Raw coal	20,908	0.7143	26.37	0.94	1.9003
Natural gas	38,931	12.143	17.20	0.99	2.1622
Liquefied petroleum gas	50,179	1.7143	15.30	0.98	3.1013

Notes. 1. The low calorific capacity of 1 kg standard coal (1 kgce) is equal to that of 29307 kJ fuel. 2. The average low calorific capacity and conversion factor of standard coal are from the *General Rules for Calculating Comprehensive Energy Consumption (GB/T 2589–2008*). The CO_2_ content per unit calorific value and Oxidation rate of CO_2_ are from Guidelines for Preparing Provincial Greenhouse Gas Inventories (National Development and Reform Commission No. [2011] 1041). 3. CO_2_ emission factor = Average low calorific capacity × Conversion factor of standard coal × CO_2_ content per unit calorific value × Oxidation rate of CO_2_.

**Table 5:** Regional division of China’s power grid.

Region	Provinces and Cities
Northern China	Beiijng City, Tianjin City, Hebei Province, Shanxi Province, Shandong Province, Inner Mongolia Autonomous Region
Northeast China	Liaoning Province, Jilin Province, Heilongjiang Province
Eastern China	Shanghai City, Jiangsu Province, Zhejiang Province, Anhui Province, Fujian Province
Central China	Henan Province, Hubei Province, Hunan Province, Jiangxi Province, Sichuan Province, Chongqing City
Northwest China	Shaanxi Province, Gansu Province, Qinghai Province, Ningxia Autonomous Region, Xinjiang Autonomous Region
Southern Region	Guangdong Province, Guangxi Zhuang Autonomous Region, Yunnan Province, Guizhou Province, Hainan Province

**Table 6:** CO_2_ emission factors of each regional grid from 2006–2019.

Year	CO_2_ Emission Factors (kgCO_2_/Kwh)					
	Northern China	Northeast China	Eastern China	Central China	Northwest China	Southern Region
2006	0.9825	1.0045	0.8640	0.9445	0.8410	0.7784
2007	1.0302	1.0517	0.9047	0.9746	0.8498	0.8434
2008	0.9928	1.0314	0.8888	0.9735	0.8712	0.8712
2009	0.8935	0.9267	0.7826	0.8529	0.8340	0.7880
2010	0.8704	0.9097	0.7691	0.7707	0.8413	0.7134
2011	0.8114	0.8420	0.7495	0.7244	0.7926	0.6323
2012	0.7980	0.8520	0.7567	0.7339	0.7656	0.6568
2013	0.8039	0.8619	0.7613	0.7385	0.7418	0.6496
2014	0.7995	0.8409	0.7478	0.7231	0.7045	0.6775
2015	0.7598	0.7803	0.7029	0.6508	0.6310	0.6304
2016	0.7253	0.7798	0.6785	0.6150	0.6392	0.5874
2017	0.7129	0.7196	0.6485	0.6063	0.6194	0.5422
2018	0.7080	0.6778	0.5886	0.5714	0.6430	0.5029
2019	0.7119	0.6613	0.5896	0.5721	0.6665	0.5089

**Table 7:** The regression result of CO_2_ emissions and per capital GDP, 2006–2019.

Variable	Coefficients	z-Statistic	Prob.
lng	3.032	6.40	0.000
lng^2^	−0.651	−4.38	0.000

**Table 8:** Two-dimensional decoupling states and dynamic path of cities in the YRB, 2006–2019.

City	Decoupling State			Dynamic Path	Total Score	Rank	Type
	2006–2010	2010–2015	2015–2019				
Zibo	WD-HE	SD-HE	WD-HE	3-4-3	10	1	I
Lanzhou	SD-ME	SD-ME	WD-HE	3-3-3	9	2	I
Sanmenxia	SD-ME	SD-HE	END-HE	3-4-2	9	3	I
Jinan	WD-HE	SD-HE	END-HE	3-4-2	9	4	I
Xining	SD-ME	WD-ME	WD-HE	3-2-3	8	5	II
Shizuishan	WD-ME	SD-ME	WD-HE	2-3-3	8	6	II
Hohhot	END-HE	WD-HE	WD-HE	2-3-3	8	7	II
Baotou	WD-HE	END-HE	WD-HE	3-2-3	8	8	II
Wuhai	WD-HE	WD-HE	END-HE	3-3-2	8	9	II
Ordos	END-HE	SD-HE	END-HE	2-4-2	8	10	II
Taiyuan	WD-ME	WD-HE	WD-HE	2-3-3	8	11	II
Luoyang	END-ME	SD-HE	WD-HE	1-4-3	8	12	II
Dongying	WD-HE	WD-HE	END-HE	3-3-2	8	13	II
Taian	SD-ME	SD-ME	END-HE	3-3-2	8	14	II
Baiyin	WD-ME	SD-ME	WD-ME	2-3-2	7	15	II
Wuwei	SD-ME	SD-ME	END-ME	3-3-1	7	16	II
Datong	SD-ME	WD-ME	WD-ME	3-2-2	7	17	II
Changzhi	WD-ME	SD-ME	WD-ME	2-3-2	7	18	II
Jincheng	WD-ME	WD-ME	WD-HE	2-2-3	7	19	II
Yuncheng	WD-ME	SD-ME	WD-ME	2-3-2	7	20	II
Linfen	SD-ME	WD-ME	WD-ME	3-2-2	7	21	II
Xi’an	WD-ME	WD-HE	END-HE	2-3-2	7	22	II
Baoji	WD-ME	WD-ME	WD-HE	2-2-3	7	23	II
Xianyang	WD-ME	SD-ME	WD-ME	2-3-2	7	24	II
Yan’an	WD-ME	WD-ME	WD-HE	2-2-3	7	25	II
Zhengzhou	END-ME	WD-HE	WD-HE	1-3-3	7	26	II
Hebi	WD-ME	WD-ME	WD-HE	2-2-3	7	27	II
Xinxiang	WD-ME	SD-ME	WD-ME	2-3-2	7	28	II
Jiaozuo	WD-ME	WD-HE	END-HE	2-3-2	7	29	II
Yinchuan	WD-ME	SD-HE	END-HE	2-2-2	6	30	II
Bayannao	WD-ME	WD-ME	END-HE	2-2-2	6	31	II
Yangquan	WD-ME	WD-ME	WD-ME	2-2-2	6	32	II
Luliang	WD-ME	SD-ME	END-ME	2-3-1	6	33	II
Tongchuan	WD-ME	SD-ME	END-ME	2-3-1	6	34	II
Kaifeng	WD-ME	WD-ME	WD-ME	2-2-2	6	35	II
Anyang	WD-ME	WD-ME	WD-ME	2-2-2	6	36	II
Jining	WD-ME	WD-ME	END-HE	2-2-2	6	37	II
Dezhou	WD-ME	WD-ME	END-HE	2-2-2	6	38	II
Binzhou	WD-ME	END-HE	END-HE	2-2-2	6	39	II
Pingliang	SD-LE	WD-ME	END-ME	2-2-1	5	40	II
Wuzhong	END-ME	SD-ME	END-ME	1-3-1	5	41	II
Weinan	WD-LE	WD-ME	WD-ME	1-2-2	5	42	II
Puyang	WD-ME	WD-ME	END-ME	2-2-1	5	43	II
Liaocheng	END-ME	WD-ME	END-HE	1-2-2	5	44	II
Zhongwei	SD-LE	END-ME	END-ME	2-1-1	4	45	III
Ulanqab	END-ME	WD-ME	END-ME	1-2-1	4	46	III
Shuozhou	END-ME	END-ME	WD-ME	1-1-2	4	47	III
Xinzhou	END-LE	WD-ME	WD-ME	0-2-2	4	48	III
Yulin	END-ME	END-ME	END-HE	1-1-2	4	49	III
Shangluo	WD-LE	WD-ME	END-ME	1-2-1	4	50	III
Tianshui	WD-LE	WD-LE	END-ME	1-1-1	3	51	III
Qingyang	WD-LE	END-ME	END-ME	1-1-1	3	52	III
Dingxi	END-LE	SD-LE	WD-LE	0-2-1	3	53	III
Jinzhong	END-ME	END-ME	END-ME	1-1-1	3	54	III
Heze	END-LE	WD-ME	END-ME	0-2-1	3	55	III
Longnan	WD-LE	END-LE	END-ME	1-0-1	2	56	III
Guyuan	END-LE	END-LE	WD-ME	0-0-2	2	57	III

## Data Availability

The data that support the findings of this study are openly available in CNKI at http://cnki.nbsti.net/CSYDMirror/area/home/index/D26.
